# Efficacy of preoperative lymphoscintigraphy in predicting surgical outcomes of lymphaticovenous anastomosis in lower extremity lymphedema: Clinical correlations in gynecological cancer-related lymphedema

**DOI:** 10.1371/journal.pone.0296466

**Published:** 2024-01-02

**Authors:** Min Young Yoo, Kyong-Je Woo, Seo Young Kang, Byung Seok Moon, Bom Sahn Kim, Hai-Jeon Yoon

**Affiliations:** 1 Department of Nuclear Medicine, Ewha Womans University College of Medicine, Seoul, Republic of Korea; 2 Department of Nuclear Medicine, Inha University School of Medicine, Incheon, Republic of Korea; 3 Department of Plastic Surgery, Ewha Womans University College of Medicine, Seoul, Republic of Korea; Sapienza University of Rome: Universita degli Studi di Roma La Sapienza, ITALY

## Abstract

**Background:**

Lymphaticovenous anastomosis (LVA) is a promising microsurgical treatment for lower extremity lymphedema (LEL). Lymphoscintigraphy effectively assesses lower limb lymphatic systems before LVA, but its role in predicting the therapeutic outcomes of LVA is indeterminate. In this study we investigate the efficacy of preoperative lymphoscintigraphy using clinical findings to predict outcomes in gynecological cancer-related LEL patients who underwent LVA.

**Methods:**

A retrospective review was conducted on consecutive gynecological cancer patients with LEL who had undergone LVA between June 2018 and June 2021. The therapeutic efficacy was assessed by measuring the change rate of the lower extremity lymphedema index (LELi) six months after surgery. Clinical data and lymphoscintigraphic findings were analyzed to assess therapeutic efficacy of LVA.

**Results:**

Out of the 60 evaluated legs, 83.3% of the legs showed improved results after LVA. Univariable linear regression analysis revealed that higher preoperative LELi, and ovarian cancer were associated with superior LELi change rate (LC rate). Absence of dermal backflow (DBF) on lymphoscintigraphy was associated with inferior LC rate. Multivariable linear regression analysis identified ovarian cancer and higher preoperative LELi were independently correlated with favorable outcomes, while the absence of DBF was independently correlated with inferior outcomes.

**Conclusion:**

The results of this study emphasizes the effectiveness of preoperative lymphoscintigraphy, preoperative LELi, and primary malignancy as predictors of LVA outcomes in gynecological cancer-related LEL patients.

## Introduction

Lower extremity lymphedema (LEL) is a chronic condition that results from a congenital or acquired impairment in lymphatic fluid transport due to mechanical obstruction, such as after trauma or oncologic surgery [1–3].

The incidence of LEL after inguinofemoral lymph node dissection was higher (18%] than upper extremity lymphedema after axillary lymph node dissection (3%) [4, 5]. However, while there are many studies on prognostic factors in lymphedema following breast cancer treatment, research on gynecological cancer-related lymphedema is relatively understudied [6–8]. This discrepancy arises from differences in the incidence rates, with a 3–4-fold higher overall incidence of breast cancer than gynecological cancer [9]. Nevertheless, as the number of cancer patients with gynecologic cancer increases and the mortality rate decreases, the number of patients suffering from LEL is increasing [10].

Lymphaticovenous anastomosis (LVA) is a microsurgery technique that creates a bypass between submillimeter-sized lymphatic vessels and venules to improve lymphatic drainage [11]. Despite the number of studies describing the usefulness of LVA in treating lymphedema and its complications, there remains a lack of clear consensus guidelines on the indicators or outcome measurements for LVA in lymphedema surgery [3].

Leg volumetry is a useful method for evaluating the outcomes of LEL treatments because it allows for objective and quantitative assessments. Various methods are available for measuring leg volumes, such as circumference measurements and the use of imaging techniques [12, 13]. The lower extremity lymphedema index (LELi), which considers the circumferences of the leg alongside the patient’s body mass index (BMI), has been developed for body type-corrected leg volume evaluation [12, 14]. The LELi has been found to be more effective in evaluating therapeutic interventions than simple circumference measurements in patients with LEL [3, 14].

Lymphoscintigraphy is an effective and minimally invasive imaging technique for evaluating the lymphatic system of the lower limbs. While previous studies suggested that lymphoscintigraphy can be useful in LVA, its application in gynecological cancer related LEL remains a topic of debate due to differing methods of imaging analysis and variations in clinical presentations [15–17].

Therefore, in this study, we investigated the effectiveness of preoperative lymphoscintigraphy and the subsequent clinical findings to predict the outcome of LVA by using the LELi in gynecological cancer-related LEL patients.

## Materials and methods

### Study design and participants

This study was conducted in accordance with the Declaration of Helsinki and was approved by the Institutional Review Board of Ewha woman’s mokdong hospital with a waiver of informed consent (IRB number 2022-09-020). Consecutive patients with LEL who had undergone LVA in our hospital between June 2018 and June 2021 were retrospectively reviewed from October 2022 to December 2022. Patients with a history of previous lymphatic surgery, or lymphedema caused by reasons other than gynecological cancer were excluded. Patients without preoperative lymphoscintigraphy were also excluded.

Patient follow-ups and clinical data collection were performed at Ewha Woman’s University Medical Center. Clinical data such as age, BMI, duration of lymphedema, primary malignancy, number of total anastomoses, episode of cellulitis, ISL stage of lymphedema, and history of radiation therapy were retrieved from the electronic medical records system. The LELi change rate (LC rate) was used for the assessment of the therapeutic efficacy of LVA using LELi measured at the preoperative workup and 6 months (± 3 months) after LVA.

### Image acquisition and analysis

Lymphoscintigraphy was acquired using a dual-head gamma camera (E.CAM; Siemens Medical Systems, Hoffman Estates, IL) equipped with a low-energy high-resolution collimator. The acquisition speed was 15 cm/min. After the patient was laid in the supine position, 0.4 mL of ^99m^Tc-phytate 37 MBq (1 mCi) was subcutaneously injected into the first and second web spaces of both feet using a 27-gauge syringe. Patients were asked to exercise between each image acquisition. Early images were obtained at 5 minutes post-injection and delayed images were obtained at 30 minutes and 1 hour. Two board-certified nuclear medicine physicians (M.Y.Y. and H.-J.Y. with more than 9 and 14 years of experience, respectively) analyzed the images in consensus and were blind to the clinical information.

Patterns of main lymphatics (trunk pattern, thigh-restricted, and lower leg-restricted), and visualization of regional lymph nodes were evaluated, according to previous reports [18]. According to the presence and extent of dermal backflow (DBF), DBF patterns were classified into the absence, small extent (dermal back flow on the thigh or lower leg only), and large extent (dermal back flow on both the thigh and lower leg) [17].

### Surgical procedure and clinical outcomes

All patients provided written informed consent prior to LVA. The surgery was performed by an experienced plastic surgeon (K.-J.W.). The surgical indication for LVA was patients with mild to moderate lymphedema for whom ICG lymphography indicated the presence of a functioning lymphatic vessels with a flow velocity grade of 2 or higher [19]. Most patients underwent lymphoscintigraphy on both legs before LVA to assess lymphedema. Lymphaticovenous shunts were created by microsurgical anastomosis using end-to-end, side-to-end, or end-to-side techniques. Lymphatic vessels were anastomosed to subcutaneous or superficial veins using 11–0 nylon (Ethicon Inc). The site of the lymphovenous shunt was determined by the surgeon. Usually, 2–4 functional lymphatic vessels with proximal obstruction were selected for LVA [18, 20]. The lower extremity circumference was measured at 7 sites (feet, ankles, 20 cm below the knee, 10 cm below the knee, knee joint, 10 cm above the knee, and 20 cm above the knee) by an assigned research assistant using an inelastic measuring tape: notably, this was more than the 5 sites previously measured in the reference paper and was performed to ensure greater sensitivity. The weight of each patient was reassessed each time the circumference of the legs was measured. LELi was calculated by recording the sum of the circumference of the squares in the 7 sites of the lower extremity and dividing it by the BMI [14]. LC rate was calculated using the difference between the LELi measured at 6 months (± 3 months) post LVA and preoperative LELi. The formula is as follows: LC rate (%) = (LELi measured at 6 months after LVA–preoperative LELi) / preoperative LELi x 100 (%). When the LC rate is ≤ 0, the surgical result was scored as improved.

### Statistical analysis

Descriptive statistical analyses were conducted for all patients enrolled in the study. To assess data distribution, the Shapiro-Wilk test was applied. When the p-value was less than 0.05, the data were represented as the median with the interquartile range (IQR), while normally distributed data were expressed as the mean ± SD. One-way analysis of variance with post hoc Bonferroni testing was performed to compare the LC rate of different clinical factors and parameters of lymphoscintigraphy. Univariable linear regression analyses were performed for each variables to identify the associations with LC rate. For the analysis, nominal variables were included as indicator or dummy variables. Multivariable linear regression analyses with stepwise forward selection methods was performed using all variable. Multicollinearity in regression was checked using the variance inflation factor index. All statistical tests were 2-sided with a significance threshold of P < 0.05. Data analyses were performed using SPSS (version 26.0; SPSS Inc, IBM Company, Somers, NY). In accordance with the journal’s guidelines, we will provide our data for independent analysis by a selected team by the Editorial Team for the purposes of additional data analysis or for the reproducibility of this study in other centers if such is requested.

## Results

### Patient characteristics

**Fig 1:**
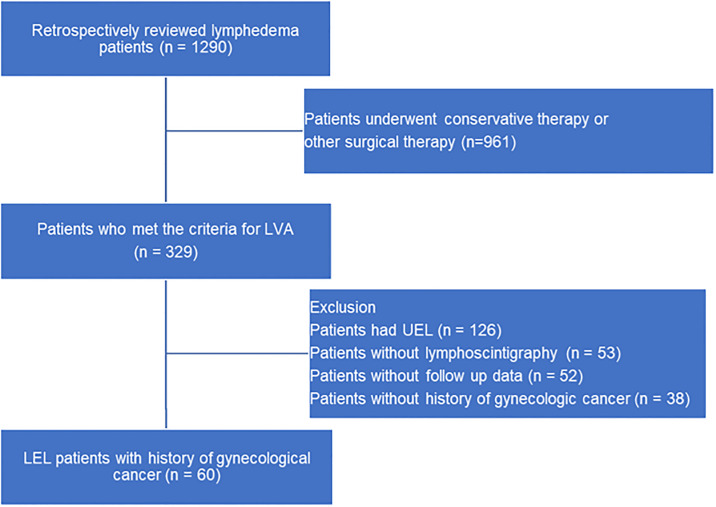
A flow chart depicting the criteria for included patients. LVA: Lymphaticovenous anastomosis, UEL: Upper extremity lymphedema, LEL: Lower extremity lymphedema.

Between June 2018 and June 2021, a total of 1290 patients with limb lymphedema visited our medical center. During this period, 329 of these patients (25.5%) presented mild to moderate lymphedema and underwent LVA. Among these patients, 60 were included in this study after excluding those without lymphoscintigraphy, without a history of gynecological cancer, with upper extremity lymphedema, and those lacking follow-up data. A flow chart depicts the selection of patients in Fig 1.

Five patients underwent LVA on both legs, and the leg with the larger LELi was included in the analysis. The demographic information of the included patients is summarized in Table 1. The most common cause of LEL was uterine cervical cancer (53.8%), which was followed by endometrial and ovarian cancers (26.7% and 20.0%, respectively). The mean age was 59.0 ± 11.0 and the median BMI was 25.0 (interquartile range [IQR], 22.0–27.0). Postoperative LELi data were collected at 6.2 months (IQR, 6.1–6.7 months) after LVA. An improvement in LC rate was observed in 83.3% of the legs. The overall mean LC rate was -4.3 ± 6.1% in all patients. Most of the patients (83.3%) had lymphedema at ISL stage 2, and 40% of them had a history of cellulitis. Lymphoscintigraphy scans showed the absence of DBF in 16.7% of cases, and visualized regional lymph nodes in 45% of the cases.

**Table 1:** Demographic details of patients.

Variables	Total patients (n = 60)
Age, y	59.0 ± 11.0
Body mass index, kg/m2	*25.0 (22.0–27.0)
ISL stage, n	
I	7 (11.7%)
II	50 (83.3%)
III	3 (5.0%)
Episode of cellulitis, n	
yes	24 (40%)
no	36 (60%)
Duration of lymphedema, months	*57.0 (13.5–108.0)
Primary malignancy, n	
Ovarian cancer	12 (20.0%)
Endometrial cancer	16 (26.7%)
Cervical cancer	32 (53.3%)
Follow-up period, months	*6.2 (6.1–6.7)
Total n. of anastomoses	*3 (3–4)
Preoperative LELi	447.6 ± 64.0
LELi change (LC) rate, %	-4.3 ± 6.1
Radiation therapy (done)	19 (31.7%)
Lymphoscintigraphic findings	
Extent of dermal backflow, n	
Absence	10 (16.7%)
Small extent	20 (33.3%)
Large extent	30 (50.0%)
Visualized regional lymph node, n	27 (45.0%)
Pattern of main lymphatics, n	
Trunk pattern	25 (41.7%)
Thigh-restricted	12 (20.0%)
Lower leg-restricted	23 (38.3%)

* Median (IQR); LELi, lower extremity lymphedema index

### Linear regression analysis for the change rate

**Fig 2:**
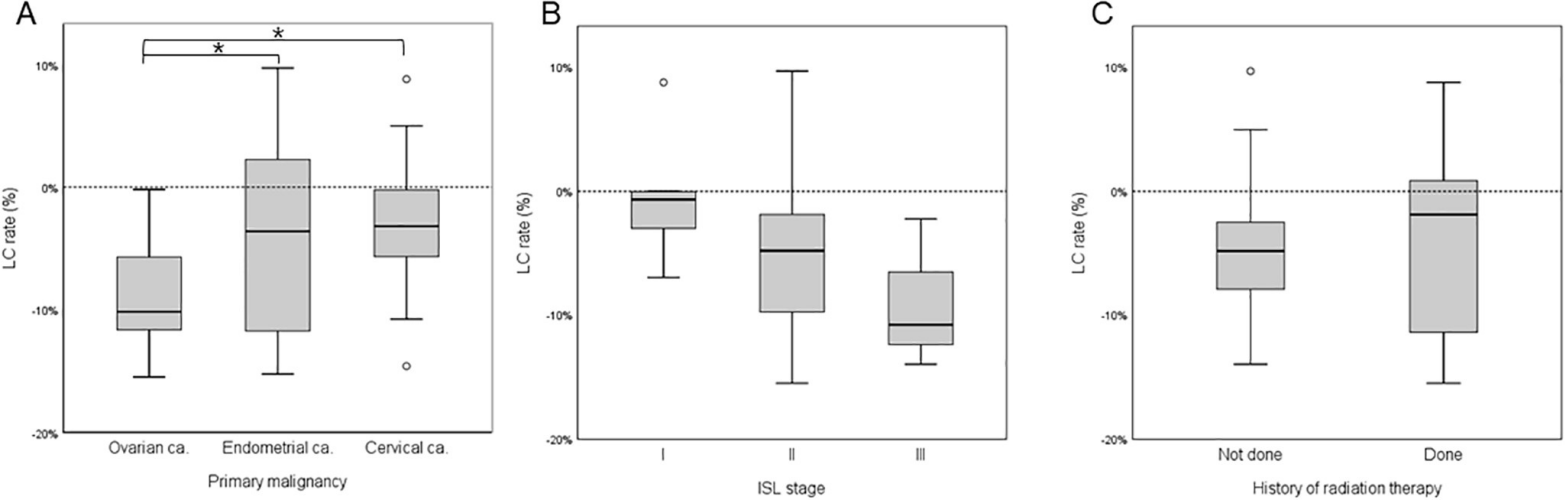
Treatment outcomes according to clinical findings. Box and whisker plots comparing LC rate (%) after LVA based on (A) primary malignancy, (B) ISL stage, and (C) history of radiation therapy. The boxes represent the interquartile distribution. Results showed significant differences in primary malignancy between ovarian cancer and other malignancy. While superior outcomes were observed with increasing ISL stage, statistical significance was not achieved. Radiation therapy did not yield significant differences in LC rate after LVA.

Univariate linear regression analysis for clinical factors using the LC rate as a dependent variable demonstrated that preoperative LELi, and primary malignancy were significant (Table 2). Endometrial cancer, cervical cancer were related to inferior outcomes of LVA, while increased preoperative LELi were related to superior outcomes. While ISL stage II and III demonstrated an association with LC rate, it did not achieve statistical significance (*p* = 0.105, and *p* = 0.053, respectively). History of radiation therapy, duration of lymphedema, episode of cellulitis, preoperative BMI, and the number of total anastomoses did not show significant association with LVA outcomes (Fig 2).

**Table 2:** Univariable regression analysis for predicting therapeutic outcome after LVA.

Variables	β	95% CI	P
Age	-0.05	-0.20–0.10	0.508
Duration of lymphedema, months	-0.01	-0.04–0.02	0.408
Preoperative BMI	-0.22	-0.69–0.25	0.346
Preoperative LELi	-0.05	-0.07 – -0.02	< 0.01
Primary malignancy			
Cervical cancer (reference)			
Endometrial cancer	4.59	-4.76–2.48	0.531
Ovarian cancer	5.73	-9.74 – -1.73	0.006
Radiation therapy			
Not performed (reference)			
Performed	1.01	-2.47–4.48	0.564
Total anastomoses, n	-1	-3.05–1.05	0.333
Extent of dermal backflow			
Large extent (reference)			
Small extent	1.46	-1.89–4.78	0.389
Absence	7.135	2.97–11.35	< 0.01
Visualization of regional lymph node			
Not seen (reference)			
Seen	2.2	-1.01–5.40	0.176
Pattern of main lymphatics			
Trunk pattern (reference)			
Thigh-restricted	-0.05	-4.39–4.30	0.983
Lower leg-restricted	-2.72	-6.29–0.86	0.133
ISL stage			
I (reference)			
II	-4.05	-8.97–0.87	0.105
III	-8.29	-16.71–0.13	0.053
Cellulitis			
Never (reference)			
Episode of cellulitis > 0	0.72	-2.58–4.02	0.663

BMI, body mass index; LELi, lower extremity lymphedema index

**Fig 3:**
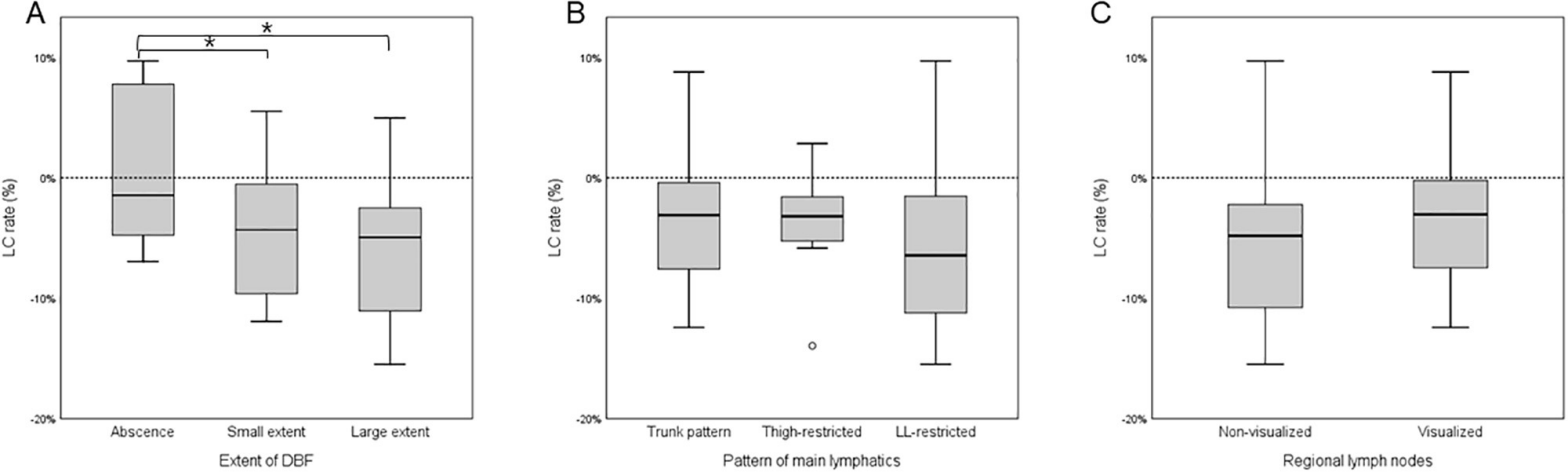
Treatment outcomes according to lymphoscintigraphy findings. Box and whisker plots comparing LC rate (%) after LVA based on (A) extent of dermal backflow (DBF), (B) patterns of main lymphatics, and (C) visualization of regional lymph nodes (LNs). The boxes represent the interquartile distribution. Significant differences were observed based on the extent of DBF, while no significant differences were found for the patterns of main lymphatics and visualization of regional lymph nodes.

In the univariable regression analysis of lymphoscintigraphy findings, only the extent of DBF demonstrated statistical significance (Fig 3A). The absence of DBF was significantly associated with inferior outcomes of LVA. The visualization of regional lymph node and patterns of main lymphatics did not demonstrate statistical significance as a predictor (Fig 3B and 3C).

**Fig 4:**
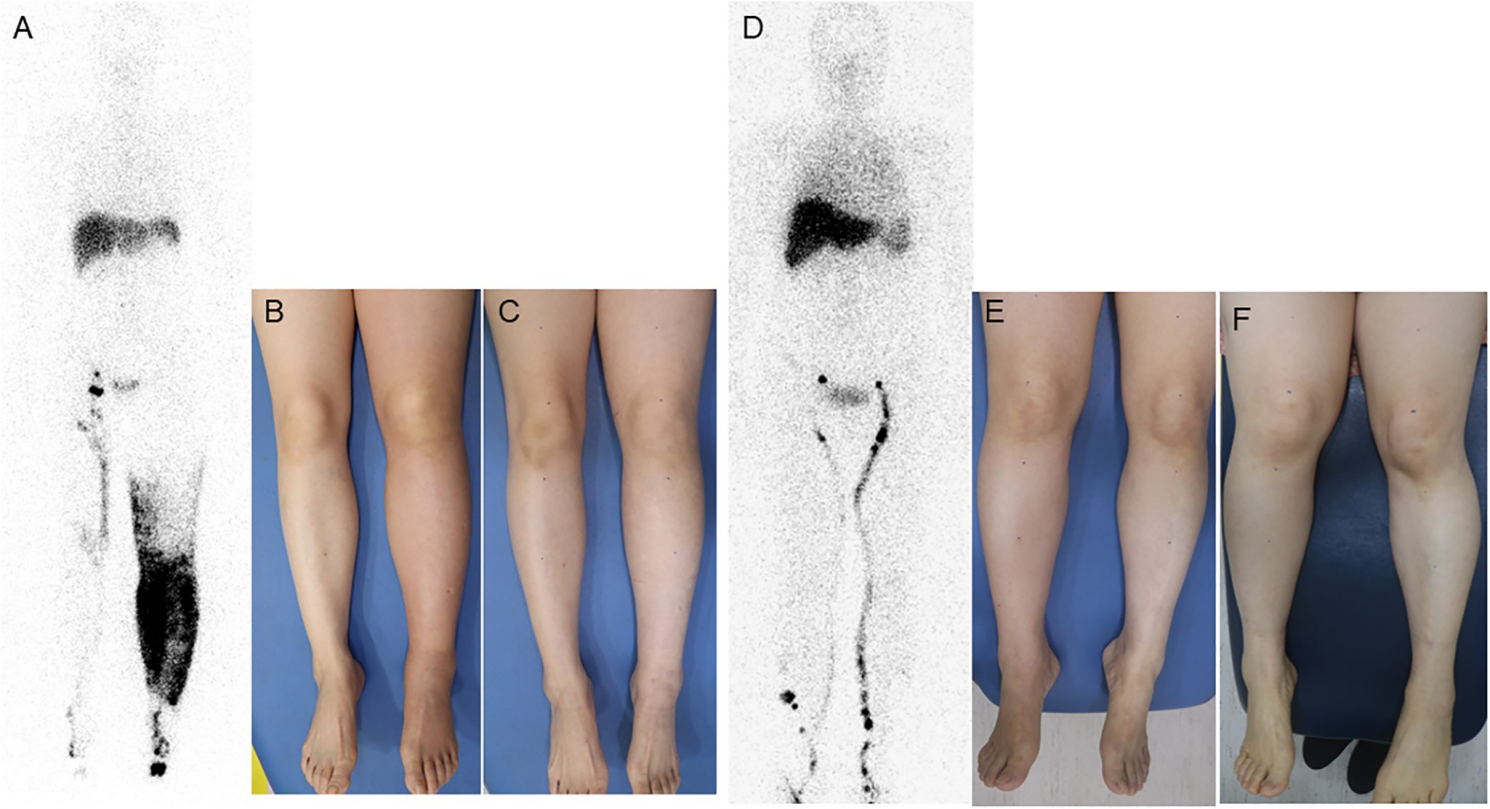
Representative case of LEL patients who underwent LVA based on lymphoscintigraphic findings. A 54-year-old woman with a history of endometrial cancer underwent radical abdominal hysterectomy (RAH) and bilateral pelvic lymph node dissection (BPLND) She had lymphedema in her left leg for the 9 months and had no history of cellulitis (A-C). (A) Lymphoscintigraphy revealed large extent of dermal backflow (DBF) on her left leg without visualization of regional lymph nodes. The main lymphatic vessels exhibited a lower-leg restricted pattern. (B) Preoperative photograph of lower extremities. The left leg presented with Stage II lymphedema with redness and pitting edema. The preoperative LELi was 446.8. (C) Postoperative photograph at 6 months. Following LVA on the left leg with three anastomoses at the saphenous, medial calf, and lateral calf, improvement in LELi was observed with enhanced redness and reduced pitting edema. After LVA, she had significant changes in LELi, with a LC rate of -13.48%. A 54-year-old woman with a history of endometrial cancer also underwent RAH and BPLND. She had lymphedema in her right leg for the 3 months and had no history of cellulitis (D-F). (D) Lymphoscintigraphy revealed no DBF with a trunk pattern of lymphatic vessels and visualized regional lymph nodes in her right leg. (E) Preoperative photograph of lower extremities. The right leg exhibited Stage II lymphedema with mild redness and pitting edema. The preoperative LELi was 440.5. (F) Postoperative photograph at 5.5 months. Four LVA anastomoses were performed at the mid lower leg, proximal lower leg, and mid-thigh. While the redness improved, pitting edema persisted. The LELi did not show improvement, with a total LC rate of 7.11%.

Among the variables, the forward stepwise linear regression selected that preoperative LELi, ovarian cancer, and absence of DBF on lymphoscintigraphy. The absence of DBF was found to be an independent factor related to the inferior outcomes of LVA (β = -4.16). Increased preoperative LELi and ovarian cancer were revealed as independent prognostic factors associated with superior outcomes after LVA (β = -0.04, and β = -5.36, respectively; Table 3). The representative cases of patients dependent on lymphoscintigraphy are depicted in Fig 4.

**Table 3:** Multiple linear regression analysis for predicting therapeutic outcome after LVA.

Variables	β	95% CI	*P*	VIF
Preoperative LELi	-0.04	-0.06 – -0.01	0.002	1.18
Ovarian cancer	-5.36	-8.57 – -2.16	0.001	1.00
Absence of dermal backflow	4.16	0.44 – 7.89	0.029	1.18

VIF, variance inflation factor; LELi, lower extremity lymphedema index

## Discussion

The absence of DBF on lymphoscintigraphy was identified as a significant independent prognostic factor associated with inferior outcomes after LVA. A increasing preoperative LELi and ovarian cancer were revealed as independent clinical prognostic factors associated with superior outcomes after LVA.

DBF on lymphoscintigraphy is characteristic finding of lymphedema that demonstrates redirection of a lymph tracer to the microlymphatics of the skin differing from the original lymphatic draining system [20, 21]. Comparative analyses of lymphoscintigraphy with ICG lymphoscintigraphy suggested a correlation between the absence of DBF and the stardust pattern which indicates progression of lymphosclerosis in NECST classification [22–24]. In a study conducted using single-photon emission computed tomography/computed tomography, it was hypothesized that if lymphatic fluid directly flows into vascular capillaries through pre-existing lymphovenous communications, DBF may not be observed [25]. Considering the findings from these studies, it is possible to explain that patients without DBF may experience reduced effectiveness after LVA [17, 26–28]. However, outcomes of LVA differ across studies according to the pattern of the main lymphatics analyses and regional lymph node visualizations. Kim et al. reported that a continuous pattern of main lymphatics (trunk pattern) and regional lymph node visualization indicated better outcomes after LVA [17]. In contrast, Cheng et al. suggested that a reduced number of regional lymph node and linear lymphatic ducts, appearing only at the distal end, were associated with favorable outcomes after LVA [28]. In this study, no significant differences in LC rates were observed based on regional lymph node visualization or the main lymphatics pattern. However, in the univariate analysis, the non-visualized regional lymph node and lower-leg restricted pattern groups exhibited relatively superior LC rate with comparable p-values (p = 0.176 and p = 0.133, respectively), align with the findings in Cheng’s study [28]. Considering that Kim’s study included cases of both of upper and lower extremity lymphedema, the observed similarities between the Cheng’s study and this study may suggest potential differences in LVA prognostic factors between upper and lower extremity lymphedema. Further research is warranted to validate these potential distinctions.

Pelvic lymph node dissection, radiation therapy, and underlying metabolic disease are recognized as risk factors for LEL in patients with gynecological cancer [29–31]. Yet, in ovarian cancer, lymph node dissection is not routinely performed unless there is node enlargement, and radiation therapy is rarely performed. Consequently, the incidence of LEL is the lowest in ovarian cancer among gynecological cancers [32]. Beyond these differences in incidence, our result demonstrated significantly worse outcomes after LVA in cervical and endometrial cancers compared to ovarian cancer. This disparity may be due to differences in the treatment approaches for each cancer type and potential underlying conditions, such as radiation therapy-induced lymphatic vessel sclerosis or metabolic diseases.

In comparision, our results also indicated that the LVA outcomes were not significantly associated to a history of radiation therapy or higher BMI, possibly attributable to the heterogeneous patient population in this study. Previous studies have reported a notable correlation between the occurrence of radiation therapy and lymphedema in cervical cancer, in contrast to endometrial cancer, and no apparent association in ovarian cancer [29, 32, 33]. Similarly, obesity significantly correlated with LEL in patients with endometrial cancer, while there was controversy in patients with cervical and ovarian cancer [29, 32, 34]. These findings underscore the complex interaction of various factors in influencing LVA outcomes across different gynecological cancer types.

Currently, surgical treatment in LEL is typically indicated for patients with clinically advanced lymphedema or uncontrolled volume unresponsive to conservative therapy [35]. Previous research suggests that complete decongestive therapy is more effective in patients with early clinical stages or smaller volume edema [36, 37]. LELi has been used to demonstrate the effectiveness of LVA in patients with LEL [38–40]. Representing the volume of retained lymphatic fluid and fibrotic tissues of the legs relative to body volume, LELi is generally regarded as associated with lymphedema severity [14]. However, our results demonstrated that patients with higher LELi exhibited better outcomes following LVA. Our internal analysis revealed minimal variance in LELi among patients in ISL stages I-II, while a significant increase in LELi was observed in patients classified as ISL stage III (Data in S1 Fig). This finding aligns with previous research indicating differences in clinical stage and pathologic state of lymphatic vessels [41]. We may hypothesize that a higher LELi correlates with a potentially more favorable response to LVA in candidates eligible for the procedure with functional lymphatic vessels, regardless of the ISL stage. These results challenge the conventional understanding of LELi as a marker of clinical severity, highlighting the imperative for additional investigation into the nuanced relationship between LELi and the prognosis of LVA.

The strengths of this study are as follows: Firstly, it employed the use of LELi, which can objectively evaluate the volumes of lymphedema, and allow for reliable comparisons in the improvement of lymphedema irrespective of the body mass index differences between subjects. Secondly, this study investigated both lymphoscintigraphy findings and other clinical factors in order to predict prognoses in clinical settings. Finally, all patients were operated on by a single surgeon, which excluded the possibility of surgical technique differences affecting prognoses.

However, there are a few limitations in this study. Firstly, in order to create uniformity in the timing of the evaluation of the surgical outcome, the follow-up period was relatively short at 6 months (±3 months), Long-term follow-up could be useful to evaluate the surgical outcomes and complications after LVA. Secondly, the outcomes of LVA were evaluated only based on the changes of the LELi without considering the presence of complications or improvements in the subjective symptoms. According to our external analysis, most of the clinical symptoms improved in all patients, and there was no significant difference between the improved and unimproved groups. Lastly, detailed surgical approaches for each cancer were not available. To elucidate the cause of the different outcomes among each different cancer type, it would be crucial to compare the surgical approaches for each cancer type in future studies.

## Conclusion

The result of this study highlights the effectiveness of preoperative lymphoscintigraphy, preoperative LEL index, and primary malignancy as independent predictors of LVA outcomes for gynecological cancer-related LEL. According to our findings, ovarian cancer and a higher LELi are significantly correlated with favorable outcomes after LVA, while the absence of DBF is significantly correlated with inferior outcomes after LVA.

## Supporting information

S1 Fig Preoperative LELi according to ISL stage. (TIF)
