# Supplementary figures and images for: Efficacy of preoperative lymphoscintigraphy in predicting surgical outcomes of lymphaticovenous anastomosis in lower extremity lymphedema: Clinical correlations in gynecological cancer-related lymphedema
Source: PLoS One. 2024 Jan 2;19(1):e0296466. doi: 10.1371/journal.pone.0296466 (PMC10760657; doi:10.1371/journal.pone.0296466)

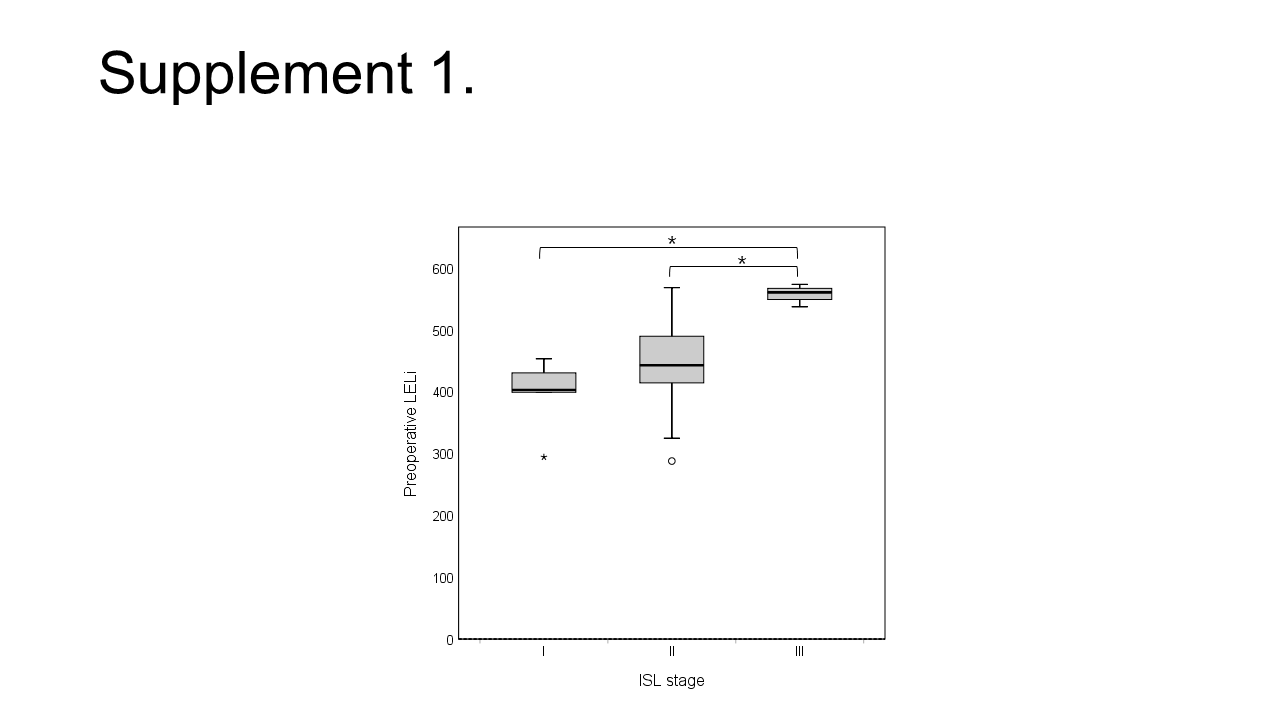

Supplement: S1 Fig — (TIF) [file pone.0296466.s001.tif]
